# Skin Diseases

**Published:** 1861-06

**Authors:** 


MARINE HOSPITAL.

### DR. ISHAM'S CLINIQUE.

Reported by STACY HEMENWAY, Student of Medicine.

B. C., aged 40, admitted April 9th; seven days previous to commencement of Prof. Isham's service.

The disease with which this patient was afflicted for more than a year, consisted of an eruption of small patches of minute vesicles about the size of a pin's head, each surrounded by a shining red areola, and in places, assuming the character of a papular eruption caused by the scratching of the patient, to allay the intolerable itching at night, when warm in bed. This eruption covered the extremities and was chiefly confined to them. In places, a laminated crust was formed over the vesicles from the exuded matter; in others it was excoriated, and successive crops seemed to appear.

His treatment had been the internal use of Fowler's Solution, and the local application of equal parts of Citrine

Ointment, and Simple Cerate. This was changed on the 17th, to the following:

℞

Zinci Oxidi, ʒiss

Amyli, ʒij

Glycerin, f℥j

Chloroform, f℥ss

M. S. To be applied to the affected parts at night; also the skin to be kept saturated during the day time with a solution of Bicarbonate of Soda (℥ss to Oj of water) through the medium of cloths applied to the parts and covered with oil silk so as to prevent evaporation. The internal use of the following was also prescribed:

℞
Magnes. Sulph.
Pot. Bitart., āā ℥j
Aquæ, Oj
M. S. A wine glass full every hour.

April 19th, condition of patient much improved. The indurated parts have begun to soften, and the skin begins to present a healthy appearance; the itching is entirely controlled by the treatment.

He was directed to take internally a solution of Pot. Iodidi ʒj to f℥iv of water in tablespoonful doses, every three hours, and to continue the external treatment.

Monday, April 29th.—Discharged completely cured.

It was remarked that in hospitals when the treatment can be enforced and strictly followed, the results of such cases were very satisfactory, but that in private practice it was a matter of the greatest difficulty, to persuade a patient to keep sufficiently quiet to secure a proper application of the lotion, and still more difficult to impress upon him the idea that constant moisture is necessary; hence the cure is frequently begun but never completed.

To avoid the irritating effects of "greasy" applications, Prof. Isham is accustomed to prescribe the use of sulphate of zinc, combined as directed in the formula above, in which the starch with the Glycerine makes an emulsion which is not "heating" as the usual ointments are, and of which patients complain, whilst the chloroform allays the hyperæsthesia of the skin. This is applied with friction by the hand; but any treatment is useless unless the patient avoids excitement of the circulation, and wears next to the skin some non-irritating and loose fitting garments.

He also stated that in his experience, the use of Citrine Ointment in such cases seemed to aggravate the disorder.

